# Consistency and Construct Validity of the Five-Level System for Risk Communication Using Static and Dynamic Tools: An Investigation Using the Static-99R and VRS-SO

**DOI:** 10.1177/10731911211061300

**Published:** 2021-12-14

**Authors:** Neil R. Hogan, Mark E. Olver

**Affiliations:** 1 Integrated Threat and Risk Assessment Centre, Alberta Ministry of Justice and Solicitor General, Edmonton, Alberta, Canada; 2 University of Saskatchewan, Saskatoon, Canada

**Keywords:** sexual violence, risk communication, VRS-SO, Static-99R, dynamic risk, psychologically meaningful risk factors

## Abstract

This study examined the Council of State Governments’ five-level system for risk communication, as applied to the Static-99R and Violence Risk Scale–Sexual Offense Version (VRS-SO). Aims of the system include increasing consistency in risk communication and linking risk categories to psychologically meaningful constructs. We investigated concordance between risk levels assigned by the instruments, and distributions of VRS-SO dynamic needs associated with Static-99R risk levels, among a multisite sample (*n* = 1,404) of persons who have sexually offended. Concordant categorical risk ratings were assigned in just over a third of cases, suggesting that consistency remains a concern with the system, particularly when conceptually disparate tools are applied. Densities of criminogenic needs varied widely among persons assigned the same risk level by the Static-99R and diverged from the descriptions ascribed by the system. These findings can inform clinical assessments and further refinement of the system.

Technology to assess risk for recidivism continues to evolve, reflecting empirical and practical advancements. As per Bonta and colleagues (Bonta, 1996; Bonta & Andrews, 2007), whereas first-generation techniques relied on unstructured and idiosyncratic professional judgments, subsequent generations incorporated actuarial predictors (second generation), dynamic risk factors (third generation), and explicit measures of change (fourth generation). The second generation of risk assessment tools, essentially a transition from unstructured to structured approaches, constituted a clear step forward with regard to reliability and predictive validity. That said, with regard to their ability to predict recidivism of one form or another, various tools from the second, third, and fourth generations have demonstrated generally comparable predictive efficacy (Campbell et al., 2009; Yang et al., 2010).

Risk instruments continue to proliferate (Singh et al., 2014), reflecting not only the various functions they are designed to serve (e.g., to inform frontline police operations, judicial sentencing decisions, treatment services) but also the complexity and variability inherent in concepts of recidivism. Elaborating on the latter point, Hogan et al. (2021) recently argued that the breadth of tools available to forensic evaluators reflects, at least in part, the fact that recidivism definitions encompass “behaviors ranging widely in form (e.g., sexual versus nonsexual violence), motivation (e.g., instrumental versus reactive aggression), and severity (e.g., serious threats, pushing, homicide)” (pp. 3–4). Furthermore, research suggests that the same instruments, even when applied to the same population, may vary in their relative predictive validity for different outcomes. For example, researchers observed varying patterns of predictive validity when applying a suite of five tools to: institutional violence observed over a period of weeks (Hogan & Olver, 2018), institutional violence observed over a period of years (Hogan & Olver, 2016), and community recidivism (Hogan & Olver, 2019). The nuanced findings with regard to predictive validity in risk assessment are also illustrated by meta-analytic reviews. For instance, although certain risk factors, such as pro-criminal attitudes, appear to predict offending of all kinds (Bonta & Andrews, 2017; Olver, Stockdale, & Wormith, 2014), it also appears that certain risk factors differentially predict more narrow outcomes, such as sexual violence (Hanson & Morton-Bourgon, 2005). In their review of intimate partner violence risk assessment tools, van der Put et al. (2019) observed that specialized tools, as a whole, did not outperform instruments designed for violence in general, but exceptions to this rule were also observed. Thus, the information provided by risk assessment tools is wide-ranging and varied, and their value is contingent upon the contexts and manners in which they are applied.

## Risk Communication

Risk assessment tools are used to inform and guide various high-stakes decisions, but their utility is influenced and limited by the manner in which their results are communicated. Noting the complexity inherent in the risk information described above, it is perhaps unsurprising that risk communication is itself fraught with difficulty. Categorical risk labels in particular, despite being the preferred form of risk information among many users (Kwartner et al., 2006), exhibit marked problems with consistency. It is rare for researchers to compare risk category assignments across instruments, to determine, for example, whether two tools would deem an individual high risk. The scant available data, drawn from the sexual violence literature, suggest that analogous risk instruments do not necessarily assign individuals who have sexually offended to concordant ordinal risk categories (Jung et al., 2013), nor do they necessarily produce concordant risk rankings (Barbaree et al., 2006). Even if the same label is assigned (e.g., *high risk*), professionals may vary widely in their interpretations of the label (Hilton et al., 2008). Based on such issues, some have argued in favor of limiting risk communication to statistical information (Scurich, 2018).

Unfortunately, even ostensibly objective numerical or statistical risk metrics, such as risk ratios, percentile ranks, or recidivism rates, are not universally understood and are susceptible to idiosyncratic interpretation (Varela et al., 2014). Statistical metrics are also dependent upon outcomes of interest, contexts, and samples or populations. In the case of the Level of Service/Case Management Inventory (LS/CMI; Andrews et al., 2004). for example, users select among different relative risk metrics (i.e., those that compare individuals) and absolute risk metrics (i.e., those that speak to recidivism), depending on the individual’s sex and whether they received a community-based, as opposed to a custodial, sentence. Notably, researchers have also observed differing rates of recidivism associated with particular risk categories from the same tool, depending on the sample studied (Kingston et al., 2008). Unsurprisingly, efforts to improve risk communication continue.

## The Justice Center Five-Level System

In an effort to address the shortcomings of extant risk communication practices by providing a common language, the United States’ Council of State Governments’ Justice Center developed a five-level system of risk categories (Hanson, Bourgon, et al., 2017). The five-level system provides a framework for populating and assigning risk categories on the basis of relative risk indicators (e.g., risk ratios), absolute risk indicators (e.g., recidivism rates), and psychologically meaningful risk factors. Assigned to Level I are individuals who are generally prosocial and who are not considered to pose a substantively higher risk of offending than that posed by members of the general population (<5% over 2 years). Individuals in Level II have “one or two identifiable criminogenic needs” (Hanson, Bourgon, et al., 2017, p. 7), are considered below average in terms of risk, and are expected to recidivate at rates ranging from 5% to 30% within 2 years. Level III constitutes the middle of the ascending risk distribution and comprises typical or average cases, with multiple criminogenic needs; individuals in this category are expected to recidivate at rates ranging from 30% to 49%. Persons assigned to Level IV demonstrate many and severe criminogenic needs, and are expected to recidivate at rates ranging from 50% to 84% within 2 years. At the top end of the risk distribution, individuals assigned to Level V demonstrate a high density and chronicity of most major criminogenic needs and are considered a virtual certainty to recidivate (>85% within 2 years).

Although the task of populating the five levels using statistical information has been described as “relatively simple” (Hanson, Babchishin, et al., 2017, p. 593), a number of conceptual and practical obstacles have nonetheless been identified and encountered by researchers. For instance, as part of a broad review of the system, Hogan (2021) pointed out that neither recidivism rates nor profiles of psychologically meaningful risk factors are universally applicable across circumscribed categories of offending behavior (e.g., nonsexual violence vs. voyeurism offenses). Consistent with such observations, obstacles were encountered during the first formal applications of the five-level system, as described below.

### Static-99R and the Five-Level System

The Static-99R (Helmus, Thornton, et al., 2012) is a second-generation risk assessment tool, designed to predict sexual recidivism among males with histories of formal sanctions for sexual offending. Hanson, Babchishin, and colleagues’ (2017) efforts to generate risk levels for the Static-99R constitute the first application of the five-level system to an existing risk assessment tool, as well as the first attempt to adapt the system for a specialized outcome—sexual recidivism.

The developers described their process, which utilized existing normative data obtained from a multisite sample of 2,395 males who had sexually offended, as follows. First, Level III was centered on the median score, with its upper and lower boundaries adjusted to include approximately 50% of the sample. Second, the lowest category was populated using a criterion-referenced definition, which deviated from the original five-level system to accommodate the focus on sexual recidivism. Specifically, the developers identified a group of individuals for whom sexual recidivism rates were expected to match the rate of spontaneous sexual offending among justice-involved persons with no history of such offenses (i.e., at or below 2% over 5 years). Third, Level II was defined, essentially, by accommodating those persons with scores falling between Levels I and III. Defining the highest two categories was less straightforward given the focus on sexual recidivism rates, because no group was identified that could reasonably be described as being virtually certain to reoffend, which is a defining feature of Level V in the original system. Ultimately, the developers opted to populate five total levels using criterion-referenced or relative risk indicators, but assigned labels of IVa and IVb to the highest categories while omitting Level V. Level IVb, the highest category, includes persons with an expected recidivism rate that is double that of the category below and 4 times that of Level III. Level IVa, essentially, comprises individuals falling between Level IVb and Level III.

### VRS-SO and the Five-Level System

The Violence Risk Scale–Sexual Offense Version (VRS-SO; Wong et al., 2003–2017) is a fourth-generation risk assessment instrument and treatment planning tool for persons with sexual offending histories. Updated VRS-SO risk levels (Olver et al., 2018) were developed after and were informed by those corresponding to the Static-99R, with the process constituting only the second formal application of the system. Nonetheless, a comparison reveals both consistencies and inconsistencies. As examples of consistencies between the two applications, the VRS-SO developers focused on sexual recidivism and employed 5-year risk estimates. The VRS-SO developers also relied upon existing normative data associated with the instrument, utilizing a multisite sample of 913 males who had sexually offended. However, conceptual differences between the two instruments contributed to some additional decision-points with respect to the VRS-SO. For instance, while the Static-99R produces single scores for individuals, the VRS-SO can produce multiple scores for an individual, including static, dynamic, and combined total scores, at both pretreatment and posttreatment junctures. Critically, recidivism rates and relative risk metrics (e.g., percentile ranks) associated with groups receiving the same posttreatment score on the VRS-SO can vary significantly, depending on such factors as individuals’ respective progress in treatment; the Static-99R has no mechanism to incorporate such considerations. To align their methods with those employed by the Static-99R developers, the VRS-SO developers elected to use a single set of percentile ranks, representing the midpoint between pretreatment and posttreatment ranks. It should also be noted that the VRS-SO developers generated separate risk categories for static, dynamic, and total scores, given that each metric may be used separately in various clinical applications.

Olver and colleagues (2018) further described the VRS-SO risk levels as follows. For each component of the instrument, Level I was defined as the lowest 5% of scores. Level II includes scores above those in Level I, but otherwise in the lowest quarter of the sample. Level III, the largest category, was centered on the average score and included approximately half of the sample. Level IVa includes scores in the highest quarter of the sample, excluding those in Level IVb. Finally, Level IVb was defined as the highest 5% of scores.

## Other Five-Level System Research

Developers of the two sexual violence risk instruments referenced above navigated hurdles in adopting the system by applying 5-year sexual recidivism rather than 2-year rates, by both omitting Level V and dividing Level IV into IVa and IVb (Hanson, Babchishin et al., 2017; Olver et al., 2018). Researchers (Davies et al., 2020) attempting to apply the five-level system to the Revised Violence Risk Appraisal Guide (VRAG-R; Rice et al., 2013), an actuarial tool designed to predict violent recidivism, encountered similar issues and ultimately recommended that further study be undertaken prior to formalizing the levels in relation to the instrument.

While the preceding examples pertain to specialized tools, recent research applying the system to a general risk/need tool, more closely aligned to the foundation of the system, has also illustrated some important considerations. Hogan (2021) argued that a normative sample that is itself representative of the range of risk in the population is likely a prerequisite for populating the five-level system, and two studies focused on the Level of Service Inventory–Revised (LSI-R; Andrews & Bonta, 1995) have now provided support for this suggestion. In the first case, Kroner et al. (2020) undertook an application of the system among a relatively low-risk sample of probationers; the authors were unable to populate Level V and ultimately elected to separate Level IV into Level IVa and IVb, respectively. In the second case, Kroner and Derrick (2020) encountered the opposite problem, with a higher risk correctional sample that was unsuitable for populating Level I. Thus, it appears that a need for further study remains if the five-level framework is to provide a universal, and universally understood, system for risk communication.

Readers should also note that converting risk instruments to the five-level system is not simply a matter of style or semantics—the changes have the potential to impact both correctional resources and individuals’ lives. Consider for instance, a study by Hogan and Sribney (2019) at a publicly funded outpatient program for sexual violence. The authors explored the potential impacts of the five-level system on their psychosexual assessments for treatment, which included the Static-99R and the STABLE-2007 (Hanson et al., 2007). Many of the participants in the study were referred by supervising probation officers, to determine whether to impose mandatory treatment, increase supervision levels, or impose additional restrictions. For 45% of the sample, the scores they received on these instruments which had placed them in the lowest of five possible risk categories now placed them in Level III based on the five-level system. To be clear, nothing had changed about the individuals, as their scores and their underlying risk profiles were identical. Nonetheless, the interpretation of their scores, and corresponding treatment and supervision recommendations, potentially changed substantially. Whether the updated risk categorizations associated with the five-level system constitute more accurate measures of individuals’ risk profiles is unknown.

## Present Study

Much of the impetus for developing a common language in risk assessment stems from the aforementioned problems with risk communication, such as inconsistencies in terminology, in the meaning applied to terminology, and in the conclusions derived from shared data, when comparing across risk tools. Theoretically, shared categories and shared principles for populating them should facilitate comparison and understanding across criminal justice applications. In fact, an explicit aim of the five-level system developers (Hanson, Bourgon, et al., 2017) was to provide a system of shared terminology whereby criminal justice stakeholders can “communicate about people precisely, clearly, and consistently, regardless of the jurisdiction where the assessment is conducted or the instrument that is used” (p. 12). In other words, a label of Level V is intended to carry the same meaning, regardless of the instrument from which it was derived. Whether this presumption of shared meaning is justified is an empirical question and warrants investigation precisely because risk instruments diverge widely in nature and content—differences potentially belied by a common risk language.

Although Hanson, Bourgon, and colleagues (2017) indicated that the shared meaning associated with the five risk levels should include both statistical information and profiles of psychologically meaningful characteristics, the limited research on the five-level system has generally emphasized statistical considerations. Even a recent study conducted by Olver, Kelley et al. (2020) focused on amalgamating actuarially derived risk categories from the Static-99R with psychologically and theoretically meaningful change information (i.e., treatment-related changes in risk) from the VRS-SO, largely emphasized impacts on the norm-referenced (i.e., relative risk) and criterion-referenced (i.e., recidivism outcomes and estimates) elements of the system. In contrast, relatively little attention has been paid to other critical elements of the system, such as the validity of categorical descriptions of psychologically meaningful risk profiles or treatment dosage recommendations. Indeed, having already used the Static-99R to populate the five-level system’s categories, Hanson et al. (2017) acknowledged that because they “propose a psychologically meaningful definition of risk categories, construct validity of these risk categories can be further researched” (p. 592). For the five-level system to be understood as truly universal and as communicating shared meaning, collection of evidence to substantiate underlying risk/need constructs is of critical clinical importance.

The present study is an attempt to validate the five-level system by focusing on two of its objectives: consistency in risk level assignment, and construct validity of clinical descriptions tied to risk levels. This was done by evaluating consistency in risk level assignment between established sexual violence risk instruments and by exploring construct validity via profiles of psychologically meaningful risk factors associated with assigned risk levels. More specifically, the first research objective was to assess concordance between risk levels assigned by the two instruments, the Static-99R and the VRS-SO, and by VRS-SO Static and Dynamic categories. The second research objective was to describe and compare the distributions of dynamic needs, as measured by the VRS-SO Dynamic scale, associated with Static-99R risk level assignments.

In the authors’ view, the Static-99R and VRS-SO are uniquely well suited for such a test of the five-level system. First, to the authors’ knowledge, at the time of writing these instruments represent the first, and only, instruments to formally operationalize and adopt the five-level system for applied use. In addition, while they are designed to predict the same outcome, the conceptual differences between them (i.e., second-generation vs. fourth-generation design characteristics) nonetheless provide a stern test of whether the five-level system can indeed ensure shared meaning regardless of the instrument employed. Such a test is warranted and necessary, given that the system itself does not make allowances for distinctions among the various generations of risk tools.

## Method

### Participants

This study utilized data derived from four existing samples of males who had sexually offended (pretreatment *N* = 1,490; posttreatment *N* = 1,365). All individuals were serving custodial sentences due to sexual offenses and participated in a sexual-offense-specific treatment program while incarcerated. The four non-overlapping samples included two groups of consecutive admissions to the high-intensity Clearwater treatment program administered by the Correctional Service of Canada (CSC), from 1983 to 1997 (Olver et al., 2007) and from 1997 to 2001 (Sowden & Olver, 2017), respectively; one group of participants from CSC’s National Sex Offender Program (NaSOP), including Low-, Moderate-, and High-Intensity streams, from 2000 to 2008 (Olver, Nicholaichuk, et al., 2014; Olver, Nicholaichuk et al., 2020); and one group of consecutive admissions to the Kia Marama Program in New Zealand, from 1993 to 2000 (Beggs & Grace, 2010, 2011) Treatment services provided to all four samples were based on cognitive behavior treatment (CBT) and guided by Risk-Need-Responsivity (RNR) principles (Andrews & Bonta, 2010), which indicate that the highest levels of intervention services should be reserved for the highest risk cases, that interventions should target criminogenic needs, and that services should be provided in manner that will maximize participants’ ability to benefit from them.

### Measures

#### Static-99R

The Static-99R (Helmus, Thornton, et al., 2012) is a second-generation risk assessment tool designed to predict sexual recidivism. This tool comprises 10 items, tapping aspects of the evaluee’s sexual and nonsexual offending history, as well as demographic variables pertaining to both the evaluee and the individuals against whom he has offended. Total scores range from −3 to 12 and correspond to risk categories adapted from the five-level system, as follows: Level I or Very Low (−3, −2), Level II or Below Average (−1, 0), Level III or Average (1 to 3), Level IVa or Above Average (4, 5), and Level IVb or Well Above Average (6 and higher). Meta-analytic results support its predictive validity (Helmus, Hanson, et al., 2012).

#### VRS-SO

The VRS-SO (Wong et al., 2003-2017) is both a fourth-generation risk assessment tool and a purpose-built treatment-planning tool. It was designed to assess risk of sexual violence, to identify treatment targets, and to assess changes in risk precipitated by credible change agents (e.g., formal treatment programs). The measure comprises 24 items, 7 static and 17 dynamic, that are each rated on a 4-point (0, 1, 2, 3) scale; higher scores signify higher risk for sexual violence. All dynamic items assigned a rating of 2 or 3 are considered criminogenic needs and are deemed high priorities for intervention, whereas those items assigned a rating of 0 or 1 are deemed to reflect low-risk domains that likely require little to no intervention. VRS-SO items can be summed to produce a total score ranging from 0 to 72, as well as component scores comprising only static items (ranging from 0 to 21) or only dynamic items (ranging from 0 to 51), respectively.

The VRS-SO risk categories were developed from 913 cases across the four normative samples (from the total pool of 1,490), with cases selected if they had a minimum 10 years’ community follow-up and had both pre- and posttreatment scores. Consistent with the Static-99R, VRS-SO total scores correspond to five risk categories adapted from the five-level system: Level I or Very Low (0 to 14), Level II or Below Average (15 to 23.5), Level III or Average (24 to 39.5), Level IVa or Above Average (40 to 49.5), and Level IVb or Well Above Average (50 to 72). Unique to the VRS-SO when compared with the Static-99R, dynamic total scores can be used to assign risk levels absent static scores, as follows: Level I (0 to 10.5), Level II (11 to 16.5), Level III (17 to 27.5), Level IVa (28 to 34.5), and Level IVb (35 to 51). Finally, for the VRS-SO static total scores absent dynamic scores, the following risk levels apply: Level I (0 to 1), Level II (2 to 5), Level III (6 to 12), Level IVa (13 to 15), and Level IVb (16 to 21).

As mentioned briefly above, the VRS-SO’s dynamic items are designed to evaluate changes in risk elicited through participation in formal treatment or by other credible change agents. One’s level of change on each treatment need is assessed at multiple time points, using a modified version of Prochaska et al.’s (1992) stages of change (SoC) model: precontemplation, contemplation, preparation, action, and maintenance. An operational definition is provided for each SoC for every dynamic item. When an individual is deemed to have progressed from one stage to the next, this results in a 0.5-point deduction from the pretreatment item score; additional 0.5 deductions are made when additional progress through the stages is made. It should be noted, however, that no deductions are made when individuals progress from precontemplation to contemplation because no behavioral changes are associated with this transition. Thus, in practice, the VRS-SO yields multiple scores of interest to professionals, including pretreatment scores and posttreatment scores, which together provide an indication of the individual’s baseline risk and any progress made toward ameliorating that risk.

### Procedure

Risk measures were rated by formally trained raters in each sample. In the case of the NaSOP sample, Static-99 and VRS-SO ratings were coded by service-providers in real time, consistent with a prospective study design (Static-99R scores were recoded from Static-99 scores by the researchers, reflecting an updated age item). In the other three samples, risk ratings were coded retrospectively for research purposes using institutional files. Although both pretreatment and posttreatment scores were available, pretreatment scores were utilized for key analyses, unless otherwise specified, given that the pretreatment ratings coincide with the application of Static-99R ratings. Readers should note that because risk level assignments are derived directly from scores with respect to both the Static-99R and VRS-SO, reliability between risk level assignments using a single score and the same tool was not in question in this study.

### Data Analytic Plan

To address the first research objective, cross-tabulation was completed to determine the proportion of cases assigned to each risk level by the respective instruments, the proportion of cases assigned to concordant and discrepant levels by the two instruments, and the distribution of discrepant cases across categories. Next, percentage agreement was calculated to illustrate the proportion of cases assigned to the same category by both instruments. Finally, a weighted kappa statistic was computed to evaluate agreement, incorporating the magnitude of disagreements (i.e., applying greater weight to larger disagreements) across levels (Watson & Petrie, 2010).

To address the second objective, descriptive statistics were computed to ascertain the number of psychologically meaningful risk factors, as measured by the VRS-SO Dynamic scale, demonstrated by persons assigned to each Static-99R risk level or VRS-SO Static risk level. One-way analysis of variance was also used to determine whether VRS-SO Dynamic scores varied among Static-99R risk levels. Finally, post hoc tests producing the Tukey beta statistic were used to compare mean VRS-SO Dynamic scores between pairs of Static-99R risk levels.

## Results

### Consistency

Table 1 presents the results from cross-tabulation and the percentage agreement calculations, comparing risk levels assigned by the Static-99R and VRS-SO Pretreatment Dynamic score. Overall percentage agreement was 35.2%. Closer examination indicates that cases assigned to a particular risk level by either tool were dispersed widely among the risk levels associated with its counterpart. In all but one instance (i.e., cases assigned to Level IVb assigned by the VRS-SO), cases assigned to a given risk level based on one tool were more likely than not to fall into a discrepant risk level using the other tool. In addition, many cases fell more than one risk category apart on the two tools. For example, of the 44 cases assigned to Level I by the VRS-SO Dynamic scale, 31 (70%) fell at least two risk levels higher using the Static-99R. Similarly, of the 362 cases assigned to Level IVb by the Static-99R, 126 (35%) fell at least two risk levels lower using the VRS-SO. The observed weighted kappa statistic of 0.266 (95% confidence interval [CI] = [.234, .299]) indicated fair agreement.

**Table 1.** Raw and Percentage Agreement Between Five-Level System Ordinal Risk Categories Assigned by Static-99R and VRS-SO Pretreatment Dynamic Scores (N = 1,404).

	Static-99R					Percentage agreement
VRS-SO	Level I	Level II	Level III	Level IVa	Level IVb	
Level I	2	11	27	3	1	4.5
Level II	12	46	66	27	6	29.3
Level III	27	121	233	163	119	35.1
Level IVa	2	20	87	113	136	31.6
Level IVb	1	5	20	56	100	54.9
Percentage agreement	4.5	22.7	53.8	31.2	27.6	35.2

*Note.* Highlighted values indicate correspondence between measures. VRS-SO = Violence Risk Scale–Sexual Offense Version.

These analyses were repeated comparing the VRS-SO Static and Dynamic five-level risk categories (Table 2). Similar findings were obtained as with the Static-99R with a 38.3% overall level of agreement. The patterns of discordance were generally very similar, although with some notable areas of divergence, given that the VRS-SO Static and Dynamic risk levels were independently developed from the same normative sample. Hence, a small number of Level IVb cases would emerge by design, whereas the Static-99R was simply scored on the existing sample. For instance, owing to the small number of VRS-SO Static Level IVb cases, more than half (55.6%) were assigned to the same VRS-SO Dynamic category, yet only 12% of VRS-SO Dynamic Level Ivb were assigned the same Static risk category. The highest level of agreement was observed with 56.8% of VRS-SO Dynamic Level III cases being assigned to the same Static category. Otherwise, as with Static-99R, there was considerable disparity in assigned VRS-SO Static and Dynamic risk levels (often exceeding one risk category), with the degree of disparity most marked at Level I. The observed weighted kappa statistic of 0.231 (95% CI = [.201, .260]) again indicated fair agreement overall between VRS-SO Static and Dynamic risk level.

**Table 2.** Raw and Percentage Agreement Between Five-Level System Ordinal Risk Categories Assigned by VRS-SO Static and Pretreatment Dynamic Scores (N = 1,490).

	VRS-SO Static					Percentage agreement
VRS-SO Dynamic	Level I	Level II	Level III	Level IVa	Level IVb	
Level I	4	27	15	0	0	8.7
Level II	26	65	63	5	0	40.9
Level III	44	192	392	57	5	56.8
Level IVa	5	30	252	84	15	21.8
Level IVb	1	9	109	65	25	12.0
Percentage agreement	5.0	20.1	47.2	39.8	55.6	38.3

*Note.* Highlighted values indicate correspondence between measures. VRS-SO = Violence Risk Scale–Sexual Offense Version.

Of note, and as would be expected, VRS-SO Static and Static-99R risk categories showed slightly better concordance, with observed weighted kappa of .321 (95% CI = [295, 347]) and fair levels of agreement.

### Construct Validity: Needs Associated With Static-99R Risk Levels

**Figure 1.**
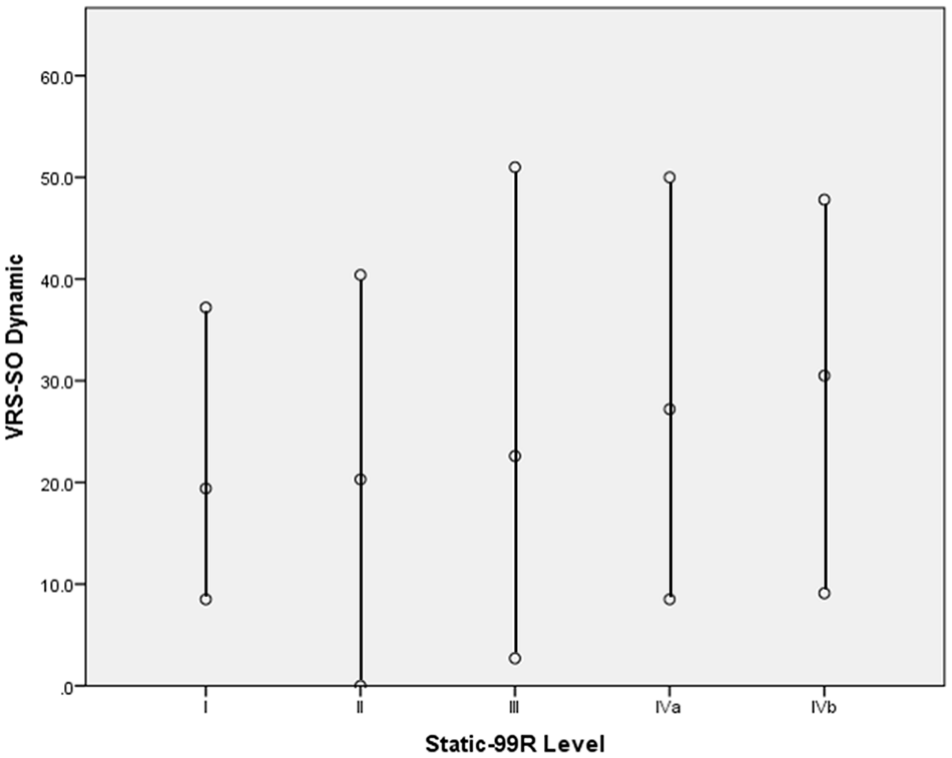
Maximum, Mean, and Minimum VRS-SO Dynamic Scores (Pretreatment) Associated With Static-99R Risk Levels. *Note.* VRS-SO = Violence Risk Scale–Sexual Offense Version.

Table 3 presents the mean VRS-SO Dynamic scale total score, as well as the minimum and maximum scores obtained by individuals assigned to each Static-99R risk level; Figure 1 also provides a visual depiction of the overlapping ranges of scores observed in each risk level. Readers may recall from the preceding methods discussion that scores of 2 points or 3 points on a particular item reflect domains identified as treatment needs. Thus, the results may be interpreted as follows: persons assigned to Level I by the Static-99R at their pretreatment assessment demonstrated a range from approximately 2–3 treatment needs up to 12–17 treatment needs; this range in totals reflects the fact that scores may reflect a greater number of items assigned scores of 2 points (but with a maximum of 17 needs, based on the total number of items) or a lesser number of items assigned scores of 3 points. Similarly, persons assigned to Level III by the Static-99R demonstrated a possible range of approximately 1 treatment need, up to a maximum of 17, reflecting a 3-point rating on every item on the scale.

**Table 3.** VRS-SO Dynamic Scores Associated With Static-99R-Assigned Risk Levels.

Static-99R level	VRS-SO dynamic score (pretreatment)				
	*n*	*M*	*SD*	Minimum	Maximum
I	44	19.4	6.0	8.5	37.2
II	203	20.3	6.8	0.0	40.4
III	433	22.6	7.5	2.7	51.0
IVa	362	27.2	7.3	8.5	50.0
IVb	362	30.5	6.8	9.1	47.8
Static-99R level	VRS-SO dynamic score (posttreatment)				
	*n*	*M*	*SD*	Minimum	Maximum
I	36	16.1	5.5	7.8	29.8
II	170	16.5	6.3	3.2	40.0
III	406	18.7	7.3	2.7	51.0
IVa	342	23.4	7.2	6.4	50.0
IVb	348	26.7	7.0	7.5	46.8

*Note.* Pretreatment *N* = 1,404; posttreatment *N* = 1,302. VRS-SO = Violence Risk Scale–Sexual Offense Version.

One-way analysis of variance was conducted to investigate whether VRS-SO Dynamic scores varied among Static-99R risk levels. The results indicated that the VRS-SO scores did differ among Static-99R risk levels at pretreatment, *F*(4, 1399) = 103.06, *p <* .001, and at posttreatment, *F*(4, 1297) = 95.71, *p* < .001. However, Tukey’s post hoc tests indicated that the mean VRS-SO Dynamic scores were not significantly different between Levels I and II, at either pretreatment or posttreatment.

When the analyses were repeated for VRS-SO Static and Dynamic scales (Table 4), a similar pattern was found. The mean VRS-SO Dynamic score increased with each successive increase in Static risk level, with the degree of separation by risk levels being significant for both pretreatment, *F*(4, 1489) = 115.63, *p* < .001, and posttreatment, *F*(4, 1360) = 103.22, *p* < .001, Dynamic scores. Again, results of Tukey’s beta post hoc multiple comparisons demonstrated VRS-SO Dynamic scores (pre and post) were significantly different between all but VRS-SO Static Levels I and II.

**Table 4.** VRS-SO Dynamic Scores Associated With VRS-SO Static Assigned Risk Levels.

VRS-SO Static level	VRS-SO Dynamic score (pretreatment)				
	*n*	*M*	*SD*	Minimum	Maximum
I	80	19.2	6.3	7.4	40.4
II	323	20.3	6.8	0.0	39.0
III	831	26.4	7.5	0.0	51.0
IVa	211	31.3	6.7	14.5	46.8
IVb	45	35.0	6.6	20.5	50.0
VRS-SO Static level	VRS-SO Dynamic score (posttreatment)				
	*n*	*M*	*SD*	Minimum	Maximum
I	69	15.6	6.4	5.3	39.3
II	289	16.6	6.3	2.1	40.0
III	761	22.5	7.4	2.7	51.0
IVa	203	27.4	7.2	12.5	46.8
IVb	43	30.9	6.9	18.1	50.0

*Note.* Pretreatment *N* = 1,490; posttreatment *N* = 1,365. VRS-SO = Violence Risk Scale–Sexual Offense Version.

## Discussion

This study evaluated the consistency and construct validity of risk levels assigned by the Justice Center Five-Level system, using two specialized sexual offense risk assessment tools in a large multisite sample of men treated for sexual offenses. Risk levels assigned by the two tools were discrepant for approximately two thirds of the sample. Furthermore, persons assigned to particular risk levels by the Static-99R or the VRS-SO Static scale varied widely with regard to their profiles of psychologically meaningful risk factors/treatment needs, as measured by the VRS-SO dynamic factors.

### Consistency and the Five-Level System

As noted in the introduction, researchers have rarely compared risk category assignments across instruments (e.g., whether two tools deem an individual high risk). The current authors are familiar with only two examples of the latter type of concordance research (Barbaree et al., 2006; Jung et al., 2013), conducted prior to the development of the five-level system, but both studies provided evidence of discordance between commonly used instruments. Furthermore, extant risk measures use different terminology and occasionally apply different meaning to the same terminology. Hanson et al. (2017) compared this state of affairs with the use of discrepant and heterogeneous measures of temperature in the 17th century, which impeded comparison and communication, and Helmus (2018) posited that the “solution to this problem is the development of a common, standardized language to describe offender risk levels” (p. 45). Whether application of the current five-level system to all risk measures can achieve the desired consistency is an empirical question, and the current findings suggest that barriers remain. Overall percentage agreement between the two measures in this study was approximately 35.2%. In other words, approximately two thirds of participants received discrepant risk ratings from the two measures. It should be noted that these discrepancies are not explained by cases falling near the boundaries of adjacent categories; approximately 18.4% of the sample were assigned to discrepant categories that fell at least two levels apart, and some discrepancies were even larger, producing a weight kappa statistic in the fair range. The overall agreement findings are generally consistent with the findings of a study conducted by Kroner and Derrick (2020) comparing a general risk/need measure with a second study-specific measure, which found an overall rate of agreement of 36.6% when using the five-kevel system. This suggests that although the five-level system provides a common language, more often than not, individuals’ risk profiles can nonetheless be described in substantively different terms depending on the specific measures applied to them.

### Construct Validity of Psychologically Meaningful Risk Profiles

In our view, the current construct validity findings provide valuable insight into the meaning of the discrepancies identified above and may elucidate potential paths forward. A well-validated second-generation actuarial risk measure, the Static-99R is moderately correlated with sexual offense recidivism (Helmus, Hanson, et al., 2012). That said, although scores on the Static-99R are also correlated with criminogenic needs, and although the Static-99R offers categorical risk ratings based on the five-level system which speak to criminogenic needs, the Static-99R is not itself a measure of criminogenic needs per se. In contrast, the VRS-SO is a fourth-generation instrument, comprising items explicitly designed to tap psychologically meaningful and dynamic risk factors, which themselves predict sexual recidivism (e.g., Beggs & Grace, 2010, 2011; Olver & Eher, 2020; Olver, Kelley et al., 2020; Olver, Nicholaichuk et al., 2014). If the risk levels assigned by the Static-99R are indeed reflective of the psychologically meaningful risk/need profiles ascribed by the system (Phenix et al., 2016), then they should correspond to risk/need profiles produced by the VRS-SO. However, the current data provided limited evidence of such correspondence. At the group level, differences in mean dynamic scores were smaller than those that the categorical descriptors suggest, and post hoc tests suggested that the levels of needs observed among Levels I and II were not significantly different. This latter finding is particularly interesting given that researchers have been unable to populate Level V with specialized tools due to issues with insufficient base rates, suggesting that there are complications to be addressed at either end of the five-level system’s risk spectrum.

Perhaps more notable, however, were the consistencies among Levels I and III. As per the Static-99R evaluators workbook (Phenix et al., 2016), Level I individuals “would not be expected to have the . . . criminogenic needs . . . typical of offenders” (p. 3), yet their scores were suggestive of a number of needs, and they demonstrated perhaps one to two fewer needs, on average, than their Level III counterparts—a group defined as “the typical offenders in the middle of the risk distribution” (p. 4). Thus, although the five-level system provides relatively clear direction with regard to treatment dosage that varies based on level, the current findings suggest that this direction should not be accepted uncritically, insofar as it is based on presumed differences in need density among levels.

Aggregate statistics, such as means and standard deviations, provide valuable information about the validity of risk measures, but they do not constitute the only psychometric properties of substantive interest for professionals. Examination of other relevant metrics, such as minimum and maximum scores, can also provide critical insights. For instance, in this study, VRS-SO Dynamic scores of the persons assigned to Level III by the Static-99R ranged from a low of 2.7 to a high of 51.0; this gulf in scores is equivalent to the difference between a person having only one known treatment need and a person maximally exhibiting all 17 possible risk factors tapped by the VRS-SO. Despite the observed discrepancy in needs, individuals at either extreme were assigned the same labels and descriptions by the Static-99R (Phenix et al., 2016), which indicates that they demonstrate “criminogenic needs in several areas, and require meaningful investments in structured programming to decrease their recidivism risk” (p. 4).

For professionals conducting assessments and distributing reports that may influence high-stakes decisions about individuals, this study raises substantive concerns. Forensic practitioners have an ethical obligation to ensure that the opinions they proffer about individuals are based on scientifically validated practices (American Psychological Association [APA], 2013), which have been appropriately applied to the particulars of the case. The APA’s (2013) Specialty Guidelines for Forensic Psychology also call upon professionals to “use assessment procedures in the manner and for the purposes that are appropriate in light of the research on or evidence of their usefulness and proper application” (p. 15). With these standards in mind, do second-generation actuarial risk tools, in and of themselves, constitute appropriate methods for assigning individuals to risk categories, as per the five-level system? On the basis of their correlations with recidivism, the use of second-generation tools to inform various decisions and match groups to graduated interventions is often not only defensible, but a professional imperative, particularly in contexts in which other structured approaches are not available. Such tools appear well suited to the statistical components of the five-level system, such as absolute and relative risk estimates. On the other hand, the current data raise concerns about second-generation tools’ relationship with the psychologically meaningful components of the system. For readers espousing greater concern for predictive accuracy than conceptual considerations, it bears mentioning that omission of psychologically meaningful risk factors can also lead to problems in calibration. Olver, Kelley et al. (2020) found that men with comparable scores on the Static-99R demonstrated different recidivism rates, depending on whether they demonstrated meaningful changes during treatment or not. Thus, although second-generation tools may demonstrate respectable predictive effects across large groups, they nonetheless omit valuable information.

A potential solution to the noted problems with regard to risk assessment is illuminated by an accumulating evidence base supporting the incremental predictive and clinical validity of existing third- and fourth-generation risk/need measures. With regard to sexual violence, notable evidence for this phenomenon can be observed among studies of the VRS-SO (e.g., Beggs & Grace, 2011; Olver et al., 2007; Olver, Beggs Christofferson et al., 2014) and the STABLE-2007 (Brankley et al., 2021). Similar findings of incremental validity for dynamic measures have been derived from studies of risk for general violence among correctional populations (e.g., Coupland & Olver, 2020; Lewis et al., 2013; Lloyd et al., 2020) and forensic psychiatric patients (e.g., de Vries Robbé et al., 2015; Hogan & Olver, 2016, 2018, 2019; Penney et al., 2016).

Returning to Hanson, Babchishin et al.’s (2017) temperature analogy for a moment, note that diverse metrics for temperature were not reconciled by simply applying shared language to various proportions of arbitrary units (e.g., the top 15%) on any given scale. Such an approach would simply have obscured meaningful differences in the various instruments, given inconsistencies in properties (e.g., minimum and maximum thresholds). Instead, efforts to standardize temperature measurement led to the pursuit of objectively meaningful criteria, such as the melting and boiling points of known compounds under particular conditions. There is perhaps not yet a direct analogue for such constructs in risk assessment. Base rates of recidivism seem an obvious choice, but they can fluctuate over time (Helmus, Hanson et al., 2012) and may vary with operational definitions and contextual factors, such as victim reporting rates and law enforcement practices (Zara & Farrington, 2016).

For risk assessment, structured measures of known criminogenic needs may provide a way forward. Standardized measures tapping criminogenic factors like substance abuse, pro-criminal attitudes, and antisocial peer networks represent constructs of interest across diverse offending populations because they predict recidivism while guiding interventions consistent with the principles of effective offender rehabilitation (Bonta & Andrews, 2017). Whereas base rates might change, offense-supportive attitudes and antisocial personality patterns make substantive contributions to a risk assessment, regardless of occasionally capricious statistics. Psychologically meaningful risk factors also retain value when assessing individuals for whom appropriate normative samples and base rates are difficult to identify or are nonexistent. Many professionals would likely agree that it is ethically defensible and prudent to target an aggressive individual’s problems with addictions or their cognitions that support violence, even before a robust actuarial table can be derived from their specific cultural group. Conversely, to refer all persons with a particular score on a second-generation tool toward treatment for problems they may or may not have (e.g., anger management), despite the availability of a more direct measure, seems difficult to justify. Fortunately, such measures may be combined directly with some second-generation instruments to assign risk levels, as is the case with the Static-99R and STABLE-2007.

Finally, it is critical to remember that problems in producing standardized and meaningful terminology with respect to psychological assessments are, of course, not unique to risk assessment. Notably, the American Academy of Clinical Neuropsychology (AACN; Guilmette et al., 2020) recently published a consensus conference statement with respect to the labeling of test scores which addressed a number of analogous issues and which may inform the efforts of other assessment professionals. For instance, the AACN group sought to replace descriptive labels that were based purely on statistical distributions linked to specific tools. They criticized such labels as being “test bound” (p. 440) and noted that they inappropriately treated test scores “as having inherent clinical meaning” (p. 440) without sufficient attention to other contextual information. They also noted a number of important conceptual considerations in refining terminology, including distinguishing between test performance and underlying pathology or abilities; in our view, such distinctions are similar to those separating correlates/predictors of recidivism from the needs and risk factors that underlie offending behavior. They also noted the importance of linking descriptive terms directly to the population of interest (e.g., persons of a particular age or sex category, or members of the population at large). Potentially of particular interest to persons pursuing standardized risk assessment language, the AACN group ultimately elected to pursue different language for different types of tests (e.g., tests with normal distributions vs. tests with non-normal distributions). Such an approach could be appropriate for risk assessment too, given large conceptual discrepancies between, for example, second-generation tools and fourth-generation tools (i.e., like the Static-99R and the VRS-SO), or tools designed to predict any recidivism versus those designed to predict the use of child sexual exploitation material. Given that the five-level system has already been modified to accommodate specialized subtypes of offending out of necessity, it seems a logical next step to explicitly acknowledge and formalize the use of parallel labels to accommodate divergent outcomes and divergent tools. Such an approach would likely ensure more precise and straightforward communication when using the new universal risk language, in keeping with sound principles established in the broader psychological assessment literature.

### Conclusions, Strengths, Limitations, and Future Directions

To the authors’ knowledge, this study represents one of the first to directly evaluate the construct validity of the psychologically meaningful elements of the Justice Center Five-Level system, as applied to a second-generation risk assessment instrument. The large and diverse multi-site sample of men who have sexually offended raises confidence in the generalizability of the findings. On the other hand, the study is limited in that it focuses on only two instruments and a specialized outcome—sexual offending. Further research will be necessary to determine whether similar phenomena arise with respect to other applications, tools, sampling populations, and offending categories.

As Hogan (2021) argued on a conceptual basis, and others have observed on an empirical basis (e.g., Davies et al., 2020; Hanson, Babchishin, et al. 2017; Olver et al., 2018), translating the generalist system to specialized offending types is not straightforward and raises a number of questions. For instance, to date, tool developers working with specialized subcategories of offending (e.g., sexual violence) have opted to populate the risk levels using the same absolute values of the original system. That is, a recidivism rate for *any* offending of approximately 40% is associated with Level III based on the original system, and a *sexual* recidivism rate of approximately 40% has been pursued to populate Level III among persons who have sexually offended. A conceptual problem with this decision is that any indicator of recidivism based on a subset will be less common than an omnibus measure; some individuals who have violently offended will also engage in other offending behaviors. To focus on only one type of offending is akin to focusing on only those offenses that occurred on Weekdays. To compensate for the relatively lower rates of sexual as opposed to general recidivism, sexual violence tool developers have altered follow-up periods from 2 to 5 years; while defensible in many respects, this approach fundamentally changes the meaning of the base rates in question. To illustrate, Hogan (2021) compared this approach to treating a yearly income of US$100 000 as equivalent to the same value over 5 years. An alternative option would be to simply adjust the expectations for recidivism proportionately downward, to reflect the relatively smaller numbers of offenses under consideration. Whether approaching the problem in this way would align the system more closely with the intended risk/need profiles is an outstanding empirical question. Additional research could also focus on whether alternative and specialized narrative descriptions are required when applying the five-level system to specialized subtypes of offending. For instance, a tool used to identify persons at risk of viewing child sexual exploitation material could require different descriptions than a tool used to identify persons at risk of violence. Finally, additional research focused on instruments that are more closely aligned conceptually, such as two fourth-generation instruments, would provide valuable insight into the potential utility of the system.

If nothing else, these findings should prompt caution among professionals and decision-makers utilizing the five-level system’s narrative descriptions of need profiles based on second-generation instruments. These descriptions are not interchangeable with those derived from third- and fourth-generation tools and may substantively misrepresent an individual’s needs, thereby potentially misguiding management resources. As such, much like Olver, Kelley and colleagues’ (2020) findings, the current results constitute a strong impetus for professionals to utilize direct and validated measures of criminogenic needs where possible. The current results also appear to lend credence to Hogan’s (2021) suggestion that when applied to second-generation tools, the five-level system may be most viable and defensible as a system of statistical labels, absent the full risk/need profiles. At minimum, these data provide a stark reminder that it is incumbent upon professions to be mindful of the strengths and limitations of their assessment procedures when offering opinions about people that inform high-stakes decisions.
